# Biological Treatments and Target Therapies for Pediatric Respiratory Medicine: Not Only Asthma

**DOI:** 10.3389/fped.2022.837667

**Published:** 2022-02-15

**Authors:** Sergio Ghirardo, Michele Mazzolai, Antonio Di Marco, Francesca Petreschi, Nicola Ullmann, Marta Lucia Ciofi degli Atti, Renato Cutrera

**Affiliations:** ^1^Pediatric Pulmonology & Respiratory Intermediate Care Unit, Academic Department of Pediatrics, Bambino Gesù Children's Hospital IRCCS, Rome, Italy; ^2^Clinical, Management and Technology Innovation Research Unit, Medical Direction, Bambino Gesù Children's Hospital IRCCS, Rome, Italy; ^3^Department of Medicine, Surgery, and Health Sciences, University of Trieste, Trieste, Italy

**Keywords:** biologics, pediatric pulmonology, asthma, innovative therapies, advances in pediatric pulmonology, molecular treatments, monoclonal antibodies, target therapies

## Abstract

We present a description of pediatric pneumology biological medications and other target therapies. The article aims at introducing the importance of a molecular approach to improve treatments. The first item treated was T2-High asthma and its current biological treatment and prescribing indications to propose a flow-chart to guide the clinical choice. Molecular rationales of such treatments are used to introduce a more general description of the biological and molecular approach to target therapies application. We introduce a general interpretation approach to neutrophilic asthma using the molecular plausibility one in order to propose possible future treatments mainly targeting interleukin-1 (IL-1), IL-17, IL-12, and IL-23. Indeed, cytokines can be excellent targets for several biological treatments. Downregulation of specific cytokines can be crucial in treating autoinflammatory and rheumatological diseases with a pulmonary involvement. Such conditions, although rare, should be early recognized as they can involve significant improvement with a properly targeted therapy. We face these conditions in a cherry-picking fashion picturing SAVI (STING-associated vasculopathy with onset in infancy), CANDLE (chronic atypical neutrophilic dermatosis with lipodystrophy and elevated temperature), and COPA (coat proteins alpha syndrome) syndrome pulmonary involvement. Such examples are functional to introduce molecular-based approach for patients with rare conditions. Molecular plausibility can be highly valuable in treating patients with not-approved but possibly highly effective therapies. Due to the rarity of these conditions, we stress the concept of basket trials using the example of cytokinin-directed immunosuppressive treatment. Lastly, we provide an example of augmentative therapy using the alpha1 antitrypsin deficiency as a model. In summary, the article presents a collection of the most recent achievements and some possible future developments of target therapies for pediatric pulmonary conditions.

## Introduction

The article aims at providing a comprehensive overview, although necessarily incomplete, of current biological treatments for asthma and other pulmonary conditions in children. The main focus will be set on molecular mechanisms, and in doing so, we will also discuss treatments that may be clinically applied in the next future. We searched PubMed and Scholar databases, limiting our consultation to the past 20 years and publications in English.

We consider it necessary to provide an adequate definition of biological treatment. Biopharmaceuticals are considered those drugs derived from a biological source, comprising somesthesis as well. This broad definition includes blood components, hormones, cellular therapies, and even gene therapies (1). In this, we decided to discuss only biological and target therapies that present an accurate and well-known interaction with a specific pathway in a key-lock mechanism, such as monoclonal antibodies.

## Severe Asthma and Endotypes

Approximately 4–5% of asthmatic children present a severe disease. Severe asthma is defined as the need for high doses of inhaled corticosteroids (ICS) plus a second treatment to achieve symptoms control, or the persistence of symptoms despite such treatments. Therefore, this step-up approach modulated on the symptoms severity and clinical response results sometimes unsatisfactory (2).

Asthmatic symptoms are the consequence of broncho-obstruction and airway inflammation. However, the causative mechanism could be different in each patient. On this basis, it was introduced the concept of endotype characterization, aimed at specifying the molecular pathway that underlays each phenotype (3). Therefore, patients with a poorly responsive asthma may need an *ad-hoc* therapeutic approach. The endotype is the complex of the molecular and pathobiological mechanisms leading to the disease, while the phenotype is the categorization based on its clinical features (4).

Two major endotype categories of asthma are usually considered. T-helper type 2 cell high endotype (T2-High) is characterized by a T-helper inflammatory response with IL-4 (interleukin-4) and Il-13 (interleukin-13) release. Such cytokines lead to IgE production (class E immunoglobulin) and IL-5 (interleukin-5), which are proliferative and act as survival factors for eosinophils. T2-High markers commonly considered in the clinical practice are high fractional exhaled nitric oxide (FeNO) values, and elevated eosinophils count in blood, sputum, and airways.

T-helper type 2 cell low endotype (T2-Low) is characterized by a neutrophilic or pauci-granulocytic inflammation, with normal levels of eosinophils in blood sputum and airways. IL-1, IL-8, IL-17, and Il-23 are the molecules implied in this endotype (5). Often eosinophilic, and allergic asthma overlap due to partially shared biological pathways.

## T2-High Asthma

T2-High asthma is characterized by the activation and abundance of T-helper 2 that plays a crucial role in generating and maintaining eosinophilic inflammation through IL-4, IL-5, and IL13 production (6). Th2 cells, once activated, present the epitopes of the antigen to B cells and, together with IL-4 production and CD40/CD40L co-stimulation, induce their activation, leading to plasma cells formation, isotype switching to IgE, and their production (7). IgEs in atopic patients are both soluble and linked to mastocytes membrane through FC fragment leading to mastocytes degranulation in the presence of the antigens (8). Therefore, IgE can be a possible target for asthma with the predominance of allergic components marked by IgE elevation.

Like mast cells, basophils liberate histamine and PGD2 (plated derived growth factor 2), but they produce IL-4 as well (4).

ILC2s (innate lymphoid cells of group 2) are those that respond to DAMPs (damage-associated molecular patterns) producing IL-5 and IL-13. Such response leads to eosinophilia without an allergen-specific stimulation (9). Therefore, such a pathway can be targeted, inhibiting its products IL-5 or IL-13 indirectly. This type of inflammation can be suggested by eosinophilia in the absence of IgE elevation.

IL-4, IL-13, PDGF2, histamine, eosinophils degranulation causes smooth cells contraction, hypertrophy, collagen deposition in airway walls leading to airway remodeling (4). Therefore, in the cases of mixed patterns without a clear predominance of allergic mediated T2-High inflammation, we consider a valid option the inhibition of both IL-4 and IL-13.

## T2-Low Asthma

Nowadays T2-High endotype of asthma can be treated with several possible biological therapies, hitting different targets in the T2-High inflammation cascade. On the contrary, T2-Low asthma endotype does not present any approved biological treatment right now. To date there are no approved biological treatments for the T2-Low asthma endotype (10). This dichotomic classification (T2-High and T2-Low) is still useful although highly simplified.

Endo-typing of asthma went beyond T2-High and T2-Low inflammation revealing a more complex reality than the dual system previously considered mainstream. Several factors contribute to defining the asthma endotype of each patient, and they can be classified on the most predominant inflammatory process or the most prevalent inflammatory cell. Grouping these entities, 4 types of inflammation were defined: allergic eosinophilic asthma, non-allergic eosinophilic asthma, non-allergic paucigranulocytic asthma, and neutrophilic inflammation. It should be stressed that these four forms of inflammation should not be considered mutually exclusive and that eosinophilic asthma may overlap with the neutrophilic one, forming a mixed complex inflammation that is more frequent in adulthood (11).

## T2-High Treatments

Nowadays, the T2-High endotype of asthma has several possible biological therapies, hitting different targets in the T2-High inflammation cascade.

**Figure 1:**
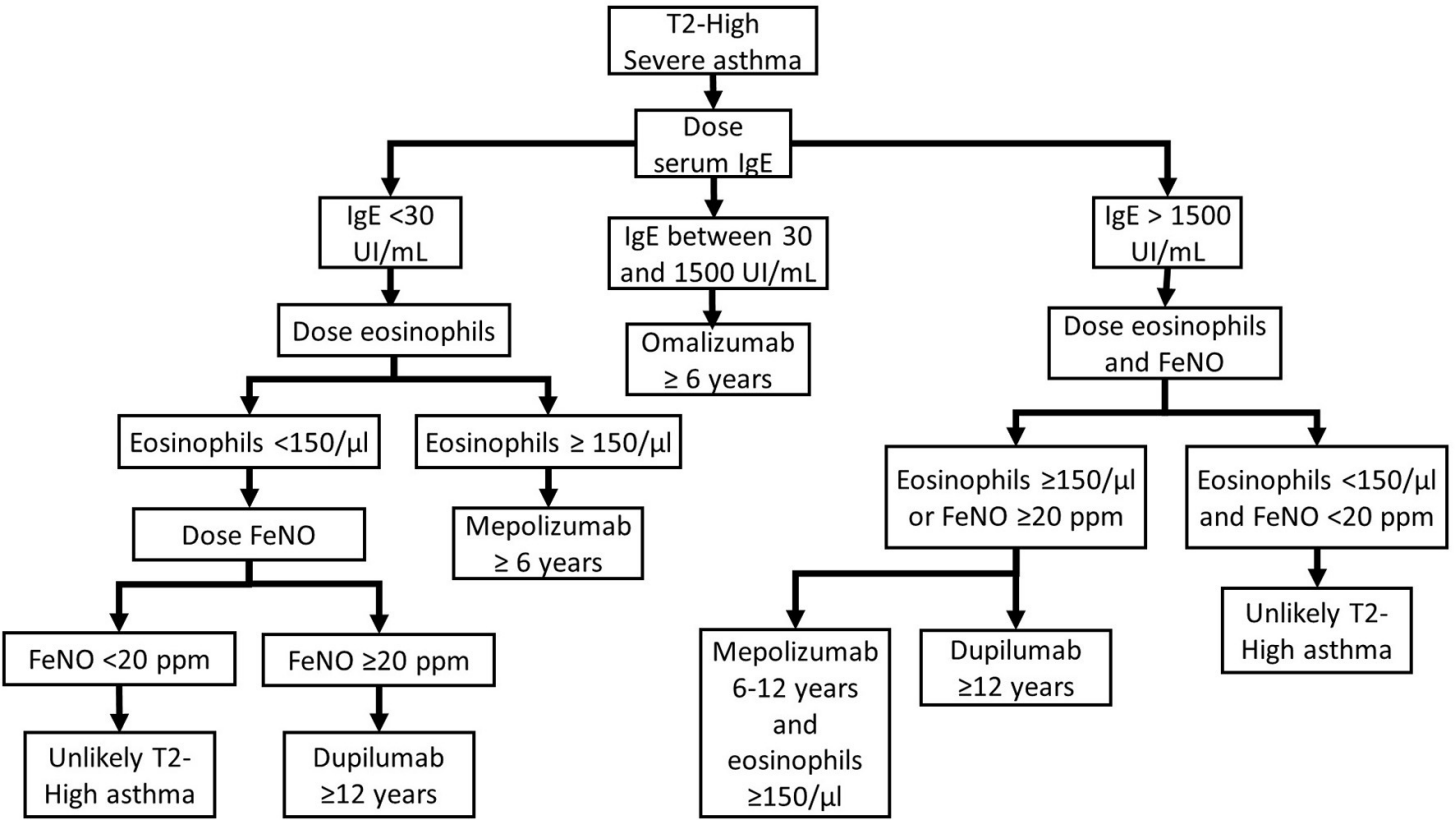
T2-High asthma biologic treatment flow-chart.

Even if there are no studies comparing the different biologic therapies for the T2-High endotype of asthma, the patient's characteristics and drug features could help choose the most appropriate treatment for each specific case. We suggest a possible flow-chart to help the choice between biological treatments in T2-High patients (Figure 1).

### Anti IgE Treatment

Omalizumab was the first biological treatment approved for the treatment of severe asthma, and it is currently recognized for the treatment of children 6 years or older. Omalizumab is a humanized IgG1 monoclonal antibody administered subcutaneously every 14–28 days. Omalizumab binds free IgE, preventing IgE from activating their receptor on the mast cells and basophils, thus reducing inflammatory molecules release. Considering its action, Omalizumab is used in patients affected by severe asthma with confirmed allergic sensitization. Omalizumab showed to reduce the number of exacerbations and the dose of inhaled corticosteroids. Previous studies on adults showed a better efficacy in patients with high FeNo, periostin and eosinophil count values (12). The patient's IgE levels are predictive of individual response. Patients with low values of IgE will only marginally benefit from omalizumab administration, whereases extremely high values of IgE could overcome the omalizumab binding power, still leaving a high level of free IgE (2, 12, 13). Omalizumab reduces to a half the risk of a severe asthma exacerbation in patients with asthma classified as moderate to severe for a total reduction from 26 to 16% of patients having an asthma attack annually and a reduction in hospitalization rate for asthma attack from 3.1 to 0.5%. Similar results were found for children with a reduction in asthma attacks of 31% in one study, a reduction of 23.7% of days with asthma in another, and the achievement of a completely controlled asthma in 52.6% more cases in the omalizumab group compared to placebo. Adverse events were significantly fewer in the omalizumab treated group than in the control group except for local reactions (14). Dosage is based on weight and IgE levels at baseline.

### Anti IL-5 Treatment

Mepolizumab is a monoclonal antibody that binds IL-5, avoiding its activating action on eosinophils, and is approved for patients 6 years or older and with a peripheral blood eosinophil count ≥ 150/microL. Mepolizumab is administered subcutaneously every month and is currently the antibody of choice for non-allergic eosinophilic asthma, that is quite rare, especially in children. It often represents a very difficult endotype to treat, requiring frequent oral corticosteroids. Mepolizumab reduces the number of exacerbations and the need for oral corticosteroids and is particularly effective in patients with marked eosinophilia. Mepolizumab's safety and pharmacodynamic were tested in patients 6–11 years with a good safety profile (15–18). The expected reduction in annualized exacerbation rate is reported to be 69% in children with severe asthma and eosinophilic phenotype (17), higher than the reduction reported for a similar population of adolescents and adults (19). Mepolizumab dose is 100 mg every 4 weeks except for patients aged 6–11 years with <40 kg of body weight who receive 40 mg of mepolizumab every 4 weeks (18). Other dosages of mepolizumab were tested effective and safe over the age of 12 administered intravenously (75, 250, and 750 mg every 4 weeks) (20).

Reslizumab has the same action as mepolizumab. It is administered intravenously 3 mg/kg of body weight every 4 weeks and has proved to be effective in reducing the number of asthmatic exacerbations by about a half. However, reslizumab is not approved in the pediatric population (21, 22).

Benralizumab is slightly different; it binds the IL5 receptor (IL-5R) on the eosinophils and basophils surface, inducing apoptosis. Benralizumab can be prescribed in patients older than 12 years, with a peripheral blood eosinophil count ≥ 150/microL. In SIROCCO study benralizumab obtained a 55% reduction of exacerbations and has shown to be more effective in patients with a high number of exacerbations per year. Benralizumab leads to profound eosinophils reduction. Like mepolizumab, benralizumab is administered subcutaneously but every 8 weeks (23–25).

Overall, in adolescents and adults, mepolizumab, reslizumab and benralizumab present a reduction in the number of severe asthma attacks of around 50%. The safety profile is optimal with no excess of severe adverse events and a similar discontinuation rate in the placebo groups, except for reslizumab that presents slightly more discontinuations in the treatment group (25).

### Anti IL-4 and IL-13 Treatment

Dupilumab is a monoclonal antibody that binds the interleukin-4 receptor (IL-4R), inhibiting the IL-4 and IL-13 molecular pathways that present a pivotal role in the Th-2 differentiation. Dupilumab initially approved for atopic dermatitis, had recently obtained approval for its use in patients older than 12 years, with severe asthma and high FeNO or peripheral blood eosinophil count ≥ 150/microL. This biological treatment showed to reduce the asthmatic exacerbation rates and the dose of oral corticosteroids as opposed to placebo (26). Dupilumab is also effective in cases with elevated FeNo in the absence of eosinophilia, suggesting an IL-13 role in this kind of patient (27). A recent trial reported the efficacy and safety of dupilumab in moderate to severe asthma patients aged 6–11 years (28).

## Neutrophilic Asthma

Neutrophilic asthma is the most common form after the eosinophilic one. Two cytokines are commonly involved in neutrophilic asthma: IL-1 and IL-17 (11, 29, 30), possibly due to deficiency in anti-inflammatory mechanisms (31). Promisingly, IL-1 receptor antagonist (anakinra) administration reduces the rise of sputum neutrophilia in healthy adults exposed to endotoxin. Neutrophil reduction is associated with IL-6, IL-8, and IL-1β decrease (32). COVID-19 related concerns interrupted a trial investigating the administration of a single dose of anakinra immediately after the exposure to an allergen in adults affected by mild allergic asthma (https://clinicaltrials.gov/ct2/show/NCT03513458). Canakinumab was demonstrated to be effective in a similarly designed trial (33). It acts on the same pathway of anakinra, but directly locking the IL-1 with a remarkably longer half-life. Although the role of the inflammasome, and IL-1, is well recognized in acute allergic manifestations (34), allergic asthma remains mainly driven by IL-5 and IL-12. In our opinion, this evidence highlight the urge for randomized therapeutic studies outside the eosinophilic forms of asthma with inclusion and exclusion criteria focused on endotypes in adults and children.

## Anti-IL17 Treatment

As aforementioned, the IL-17 cascade is deeply involved in neutrophilic asthma (11). Technically speaking, IL-17 belongs to the family of proinflammatory cytokines highly conserved through different species that chemoattracts neutrophils and monocytes. IL-17 has a huge role in inducing and maintaining this type of inflammation, especially in autoimmune diseases, such as psoriasis and ankylosing spondylitis (35, 36). Different forms of IL-17 play different roles in the various phases of inflammation, with IL-17A prevalence as proinflammatory and IL-17F predominance during resolution (37). The different affinity for the various IL-17 receptors leads to the activation of different receptors and results in an opposite contribution of these molecules, part of the same family (38). IL-17A is produced by macrophage, mainly in response to IL23, which is fundamental for the differentiation and survival of T-helper lymphocytes-17 (Th-17) which, in turn, are pivotal in mucosal inflammation producing IL-17, IL-21, IL-22, and GM-CSF (39). Such kind of IL-17 driven inflammation is also involved in allergies and asthma (40) but it is possible to be secondary to the mucosal damage caused by T2-High inflammation. Because of this whole body of considerations, the IL-17 cascade is a remarkable target for future treatments of neutrophilic asthma, especially in children who present a high count and proliferation of Th-17 in the induced sputum (41). In September 2021, we started secukinumab in a patient affected by psoriasis who presented moderate-severe asthma with constantly low expiratory nitric oxide as a sign of non-eosinophilic asthma. After the start of the treatment, his quality of life improved, by reducing acute treatment needs (unpublished data). Secukinumab is a monoclonal antibody that binds IL17A approved for moderate to severe psoriasis, psoriatic arthritis, and some forms of spondylitis from 6 years (42).

## Anti-IL-12 and Anti-IL-23 Treatments

Inhibition of IL23 with Risankizumab was proven ineffective and even harmful in adults with severe asthma (43). Once more, in this study, the eosinophilic inflammation was prevalent and confirmed the need for further studies focused on non-eosinophilic asthma.

Only one case was published, reporting a remarkable improvement in neutrophilic asthma receiving ustekinumab for severe psoriasis. Ustekinumab works upward in the IL-17 cascade and inhibiting IL23, lowering IL-17 production and the Th-17 recruitment. Ustekinumab binds IL12 as well, determining a reduction in Th1, natural killer, and cytotoxic lymphocytes activity making ustekinumab a possible effective drug for neutrophilic asthma (30).

## Interstitial and Autoinflammatory Diseases

**Figure 2:**
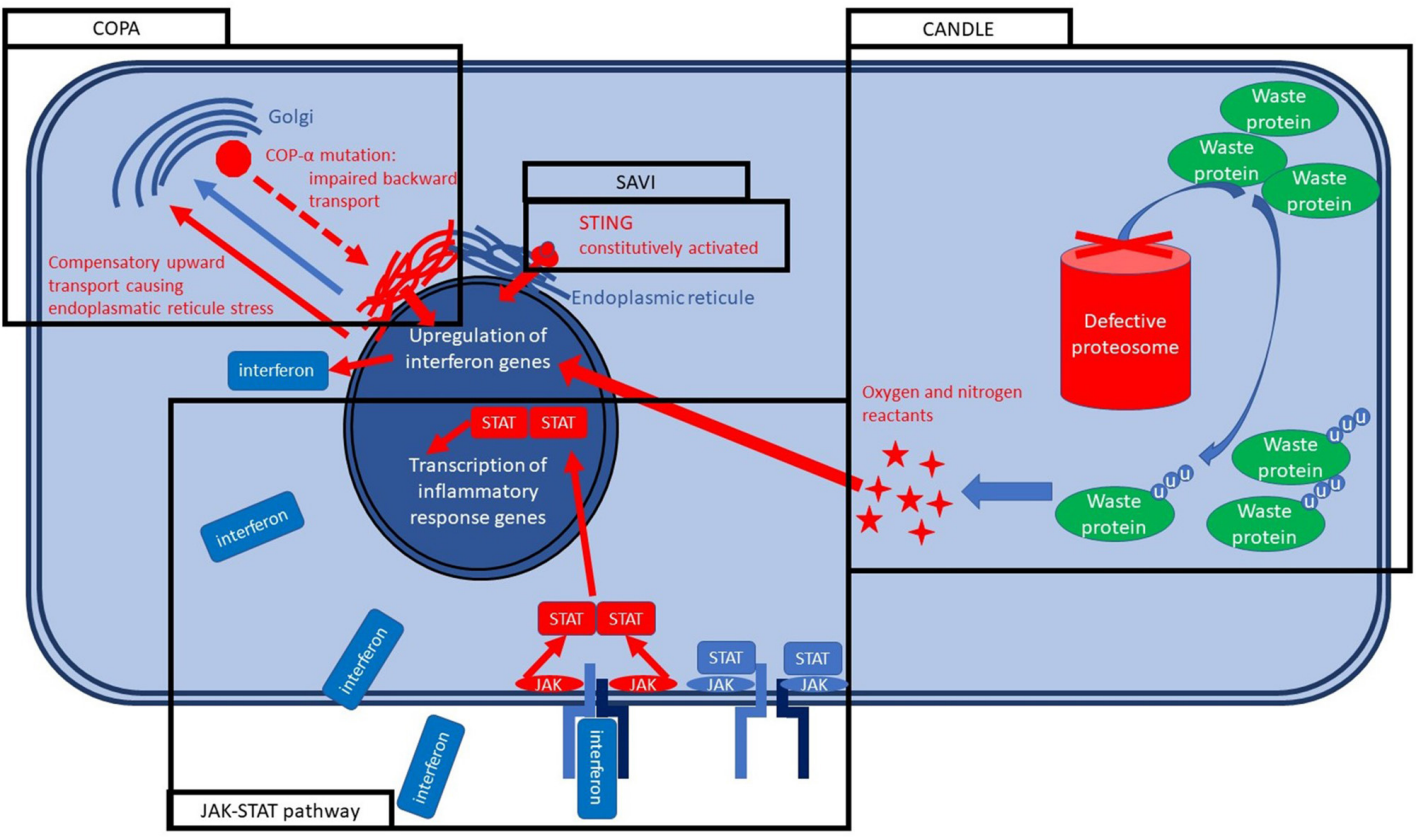
Molecular bases of SAVI, COPA, and CANDLE syndrome.

Under the classification of children's interstitial lung diseases (chILD) fall hundreds of different conditions leading to interstitial involvement and are often extremely difficult to treat. A tiny minority of chILD present an underlying genetically determined systemic autoinflammatory disease (44). As we mentioned in the previous section, a treatment directed against specific cytokines or their receptors can shut down or mitigate specific inflammation forms. We will see three different conditions causing chILD, that share high levels of type I interferon. A summary of the molecular basis of such conditions is reported in Figure 2.

## Savi Syndrome

STING (stimulator of interferon gene)-associated vasculopathy with onset in infancy (SAVI) is a systemic autoinflammatory disease caused by a mutation in the STING gene, causing its constitutive activation resulting in an autosomal dominant disease in nearly all cases. STING protein is located in the endoplasmic reticulum, and its activation is part of the early innate response to the viral infections (45) and bacterial infections, especially the intracytoplasmic ones (46). STING activation causes interferon type I releases (alpha and beta interferons) that bind the Janus Kinase (JAK) membrane receptor inducing its dimerization and activating the JAK-STAT pathway. JAK-STAT pathway has a broad action on several genes activation and transcription, determining the cellular response to interferons and other cytokines (45). SAVI syndrome is characterized by vasculitis urticaria that is usually severe, cold-sensitive, leaves scars, and is even worse on the extremities. Polyarthritis is so aggressive to induce ulcerations and, after that, autoamputations in most of the untreated cases. The pulmonary involvement is precocious, characterized by dry cough due to the chILD, and often jointly with the inflammatory status leads to a marked failure to treat (47, 48).

## Candle Syndrome

Chronic atypical neutrophilic dermatosis with lipodystrophy and elevated temperature (CANDLE), formerly known as Nakajo–Nishimura syndrome, is another monogenic condition caused by the PSMB8 gene encoding for a protein of the proteasome. PSMB8 encodes for immunoproteasome subunit β5i and, together with other mutations of the immunoproteasome, are grouped within the proteosome-associated autoinflammatory syndromes (PRAASs). PRAASs determine the ineffectiveness of the immunoproteasome with a consequent increase of old and misfolded waste proteins. Therefore, the cell responds by increasing interferons that usually activate and assemble the immunoproteasome. The lack of immunoproteasome effectiveness leads to a vicious circle in which type I interferon levels rise uncontrolled (49). CANDLE presents an autosomal recessive inheritance pattern and is clinically characterized by recurrent fever, neutrophilic dermatosis that leave purpuric scars, microcytic anemia, arthralgia leading to contractures in the long term. Lipodystrophy is usually severe and can be considered a landmark of the condition in the advanced stage of the disease (50). Pulmonary involvement is characterized by chILD and is usually less severe than for SAVI but may cause pulmonary hypertension. Early symptoms usually appear within the first weeks to 6 months of life (51).

## Copa Syndrome

Coat proteins alpha (COPA) syndrome is an autosomal dominant condition caused by a mutation in a gene encoding for a coatomer protein. More specifically, COPA participates in Coatomer Protein I (COPI) assembly, which is crucial for the backward trafficking of the proteins between the Golgi vesicles and from the Golgi apparatus to the endoplasmic reticule, leading proteins to stuck in the Golgi. An incorrect assembly of coated vesicles impairs the trafficking with a consequent intracellular stress and, therefore, interferon pathway activation, but also IL-1B and IL-6 release with Th-17 proliferation (52). Clinical features are dominated by arthritis and pulmonary fibrosis that may or may not arise before the articular symptoms and leads to progressive lung function decline. In nearly 50% of cases, pulmonary hemorrhage occurs, and it can be life-threatening. Both big and small articulations usually develop arthritis. Nephritis should always be searched and may lead to chronic kidney insufficiency. Occasionally optic neuromyelitis and avascular necrosis of the femoral head were described (53).

## Identifying a Single Target for All Three Diseases

Small molecules are usually designed to present a key-lock action, with very narrow or no effects on other pathways. Such medicines are generally obtained by full chemical synthesis and therefore are not biologics (54). Such a precise key-lock mechanism may lead to the misconception of a single extremely precise drug for each specific disease. On the contrary, the key-lock inhibition of a single molecule may be crucial for different pathways if they converge on the inhibited molecule (55). We will use the example of the three above-mentioned different genetic diseases caused by three different genes and clinically heterogeneous (SAVI, CANDLE, and COPA). All three share the type I interferon as a main standing effector of the inflammation in the final part of their cascade (56). Therefore, the inhibition of the interferon type I effector pathway was explored. Type I interferon acts through the JAK signal transducer and activation of transcription (STAT) pathway (JAK-STAT pathway) that is the effector of the cytokine receptors and several other receptors leading altogether to an extremely broad type of intracellular responses. JAK-inhibitors can specifically target a single type of JAK. In some cases, the inhibition of two JAKs type at once can be preferable as for baricitinib and ruxolitinib that inhibit both JAK1 and JAK2. Both were effectively tried in all three diseases with dramatic improvements.

## Basket Trial

This concept of inhibition of a single molecule key for several diseases went so further that recently trials were approved to test the effectiveness of one single target therapy for different diseases at once. Most of such trials are oncological ones, but IL-1inhibition was also tested in this specific study design called basket trial (57, 58).

## Augmentation Therapy

Augmentation therapy provides a homologous of a defective molecule (usually an enzyme) to keep its function within a tolerable range to avoid the progression of the disease caused by a molecule deficiency. Such type of therapy is broadly diffused in metabolic medicine but is also used for some immunodeficiencies from hypogammaglobulinemia to the Adenosine DeAminase Severe Combined Immune Deficiency (ADA-SCID). Augmentation therapy is part of the common practice of adults' pneumologists to treat alpha-1 antitrypsin deficiency. This therapy is administered intravenously weekly when serum levels are confirmed under 11 μM (80 ng/mL), and FEV1 is reduced to 35-70% of the predicted value (59) or in the presence of a rapidly evolutive condition. Alpha-1 antitrypsin deficiency is usually clinically manifested due to hepatological involvement (60) but in rare cases, patients may present even severe pulmonary emphysema already in childhood. Specific mutations, null and Z, account for low or zero serological levels of alpha-1 antitrypsin, respectively (61), leading to an earlier onset of pulmonary emphysema. Very few pediatric cases of alpha-1 antitrypsin were treated with augmentation therapy (62).

## Conclusions

Target therapies are rapidly changing pediatric pulmonology, causing a turning point in the patients' care. Such a shift in the mindset of clinical approach is ongoing and led to the need for a biologically precise diagnosis to administer the correct *ad-hoc* therapy that may be even more challenging. As a result, some of the classical randomized controlled trials designed to treat a disease may be inconclusive due to various underlying biological mechanisms. The obvious risk is the fragmentation of the study population with a consequent extreme difficulty in reaching the sample size for clinical studies exploring therapies for each biological cascade. Obtaining an adequate sample size will be even more challenging in the pediatric population. The concept of basket trial may be a possible partial answer to reach the necessary sample size for such studies.

## Data Availability Statement

The original contributions presented in the study are included in the article/supplementary material, further inquiries can be directed to the corresponding author/s.

## Author Contributions

SG, MM, AD, and FP drafted the manuscript. NU, MC, and RC reviewed the manuscript. All authors contributed to the article and approved the submitted version.

## Conflict of Interest

The authors declare that the research was conducted in the absence of any commercial or financial relationships that could be construed as a potential conflict of interest.

## Publisher's Note

All claims expressed in this article are solely those of the authors and do not necessarily represent those of their affiliated organizations, or those of the publisher, the editors and the reviewers. Any product that may be evaluated in this article, or claim that may be made by its manufacturer, is not guaranteed or endorsed by the publisher.
